# Principal Component Analysis of the Effects of Environmental Enrichment and (-)-epigallocatechin-3-gallate on Age-Associated Learning Deficits in a Mouse Model of Down Syndrome

**DOI:** 10.3389/fnbeh.2015.00330

**Published:** 2015-12-11

**Authors:** Silvina Catuara-Solarz, Jose Espinosa-Carrasco, Ionas Erb, Klaus Langohr, Cedric Notredame, Juan R. Gonzalez, Mara Dierssen

**Affiliations:** ^1^Systems Biology Program, Cellular and Systems Neurobiology, Centre for Genomic Regulation, The Barcelona Institute of Science and TechnologyBarcelona, Spain; ^2^Centre for Genomic Regulation, Universitat Pompeu FabraBarcelona, Spain; ^3^Bioinformatics and Genomics Program, Comparative Bioinformatics, Centre for Genomic Regulation, Barcelona Institute of Science and TechnologyBarcelona, Spain; ^4^Neurosciences Research Program, Human Pharmacology and Clinical Neurosciences Research Group, IMIM (Hospital del Mar Medical Research Institute)Barcelona, Spain; ^5^Department of Statistics and Operations Research, Universitat Politècnica de Catalunya/BARCELONATECHBarcelona, Spain; ^6^Centre for Research in Environmental EpidemiologyBarcelona, Spain; ^7^Centro de Investigación Biomédica en Red de Epidemiología y Salud PúblicaBarcelona, Spain; ^8^Centro de Investigación Biomédica en Red de Enfermedades RarasBarcelona, Spain

**Keywords:** Down syndrome, aging, (-)-epigallocatechin-3-gallate, Morris water maze, principal component analysis

## Abstract

Down syndrome (DS) individuals present increased risk for Alzheimer's disease (AD) neuropathology and AD-type dementia. Here, we investigated the use of green tea extracts containing (-)-epigallocatechin-3-gallate (EGCG), as co-adjuvant to enhance the effects of environmental enrichment (EE) in Ts65Dn mice, a segmental trisomy model of DS that partially mimics DS/AD pathology, at the age of initiation of cognitive decline. Classical repeated measures ANOVA showed that combined EE-EGCG treatment was more efficient than EE or EGCG alone to improve specific spatial learning related variables. Using principal component analysis (PCA) we found that several spatial learning parameters contributed similarly to a first PC and explained a large proportion of the variance among groups, thus representing a composite learning measure. This PC1 revealed that EGCG or EE alone had no significant effect. However, combined EE-EGCG significantly ameliorated learning alterations of middle age Ts65Dn mice. Interestingly, PCA revealed an increased variability along learning sessions with good and poor learners in Ts65Dn, and this stratification did not disappear upon treatments. Our results suggest that combining EE and EGCG represents a viable therapeutic approach for amelioration of age-related cognitive decline in DS, although its efficacy may vary across individuals.

## Introduction

Down syndrome (DS) is the most prevalent genetic cause of intellectual disability arising from trisomy of chromosome 21 with an incidence of approximately 1 in 1000 live births worldwide. DS affects the development and function of the central nervous system throughout life, leading to a distinctive profile of cognitive impairment and increased risk for Alzheimer's disease (AD) neuropathology. By the age of 40, almost all DS adults develop AD-like neuropathology and by the age of 55–60, around 70% develop dementia (Wilcock and Griffin, 2013). DS brains exhibit extracellular deposition of amyloid-ß (Aβ), following a fronto-striatal pattern (Wisniewski et al., 1985; Mann, 1988; Lemere et al., 1996), while hyperphosphorylated tau, in the form of neurofibrillary tangles, accumulates later in life affecting mainly the hippocampal formation, the entorhinal cortex, and the neocortex (Hof, 1995; Hyman, 1995).

So far, therapeutic interventions aimed at slowing down cognitive decline in AD such as N-methyl-D-aspartate (NMDA) receptor antagonists (memantine), anticholinesterase inhibitors (donepezil, rivastigmine, galantamine), or GABA-A antagonists have not been able to demonstrate improvements in cognitive performance in demented nor in young non-demented DS subjects (De la Torre and Dierssen, 2012). In recent years, treatment with (-)-epigallocatechin-3-gallate (EGCG), the most abundant polyphenol found in green tea, has gained attention as it has beneficial effects in AD mouse models possibly contributed by its antioxidant activity, free radical scavenging, iron chelating, anti-inflammatory effects, neuroprotection, and promotion of the non-amyloidogenic pathway of APP through ADAM10 maturation (Obregon et al., 2006; Kalfon et al., 2007; Rezai-Zadeh et al., 2008; Biasibetti et al., 2013; Kim et al., 2014). Interestingly, EGCG also has inhibitory properties on the kinase activity of DYRK1A (Bain et al., 2003; Adayev et al., 2006; Wang et al., 2012), a DS candidate whose overabundance is associated with DS neurocognitive symptoms and neurodegenerative phenotypes (Becker et al., 2014). EGCG ameliorates cognitive deficits not only in AD and DS mouse models, but also in young adults with DS (Lee et al., 2013; De la Torre et al., 2014).

Additionally, non-pharmacological therapeutic intervention, such as environmental enrichment (EE), has been successfully used in mouse models of AD (Jankowsky et al., 2005; Lazarov et al., 2005; Berardi et al., 2007; Li et al., 2013; Polito et al., 2014) and DS (Martínez-Cué et al., 2002, 2005; De la Torre et al., 2014). Interestingly, many of the effects reported for EE are similar to those observed upon EGCG treatment, such as neuroplasticity enhancement, antioxidant activity, anti-inflammatory function, neuroprotection, and promotion of the non-amyloidogenic proteolytic pathway of APP (Ickes et al., 2000; Jankowsky et al., 2005; Birch et al., 2013; Mármol et al., 2015). In fact, EE has also been shown to normalize the expression levels and the kinase activity of DYRK1A in mice overexpressing Dyrk1A and in Ts65Dn mice (Golabek et al., 2011; Pons-Espinal et al., 2013).

In the present study, we investigated the effects of combined treatment with EGCG and EE on hippocampal-dependent learning and memory, which is one of the cognitive domains most susceptible to age-associated decline and primarily affected in AD and DS (Granholm et al., 2000). To this end, we used the Ts65Dn mouse model of DS, which bears a segmental trisomy for MMU16 (syntenic region to HSA21) from Mrpl39 to Zfp295 covering APP and DYRK1A, and shows predictive validity with DS (reviewed in Dierssen, 2012) including AD-like cholinergic neuronal loss and age-associated cognitive decline (Holtzman et al., 1996; Seo and Isacson, 2005; Contestabile et al., 2006). The Ts65Dn mouse model only partially recapitulates AD pathology since it does not exhibit extracellular β-amyloid-containing plaques or neurofibrillary tangles. However, it develops other abnormal neuronal processes associated to Aβ production such as enlarged neuronal early endosomes, or increased immunoreactivity for markers of endosome fusion and recycling (Cataldo et al., 2003) that lead to alterations in NGF retrograde transport from the hippocampus to the BF (Salehi et al., 2006). Thus, it is an adequate model to investigate potential therapeutic interventions to tackle some of the common pathogenic mechanisms between DS and AD.

We assessed the effects of the treatments on spatial learning and memory performance in the Morris water maze by using classical single-variate measures such as escape latency, Gallagher index or thigmotaxis. However, learning is a process that involves the orchestration of a myriad of cognitive and behavioral outcomes, and a single variable cannot capture its essence. Learning is also measured by variables that are themselves influenced by different factors. Only under certain conditions will these measures provide the information they were designed for (e.g., latency is a good measure if all animals have the same speed, or the time spent in the periphery if it is associated with thigmotactic behavior). Such idealizations are hard to justify in an experimental context where high variability between subjects is the rule, not the exception. PCA allowed to assess the learning impairment in Ts65Dn mice and the effects of EE, EGCG, and EE-EGCG treatments in a less variable-dependent manner. We examined the relative contribution of seven behavioral variables to the variance in the data obtained from multiple water maze measurements. We identified two composite variables that together explained 86% of the variance among groups: one related to learning, and the other one mainly measuring the component of swimming speed that is not target-directed.

## Materials and methods

### Ts65Dn mouse colony

Ts65Dn and wild type (WT) littermate mice were obtained through repeated crossings of B6EiC3Sn a/A-Ts(17^16^)65Dn (Ts65Dn) females to B6C3F1/J males purchased from The Jackson Laboratory (Bar Harbor, ME). The mouse colony was bred in the Animal Facilities of the Barcelona Biomedical Research Park (PRBB, Barcelona, Spain, EU). Mice were housed in standard or enriched conditions (see below) under a 12:12 h light–dark schedule (lights on at 8:00 a.m.) in controlled environmental conditions of humidity (60%) and temperature (22 ± 2°C) with food and water *ad libitum*. Both the Ts65Dn and euploid mice were genotyped by qPCR, in accordance with the Jackson laboratories protocol (https://www.jax.org/research-and-faculty/tools/cytogenetic-and-down-syndrome-models-resource/protocols/cytogenic-qpcr-protocol).

Experiments were conducted using 5–6 months old female mice. This age represents the starting point of gradual cognitive decline (Granholm et al., 2000) and we used females since Ts65Dn males show high levels of stress in EE conditions that could mask the effect of the treatments (Martínez-Cué et al., 2002). All animal procedures met the guidelines of European Community Directive 2010/63/EU and the local guidelines (Real Decreto 53/2013) and were approved by the Local Ethics Committee (Comité Ético de Experimentación Animal del PRBB (CEEA-PRBB); procedure numbers MDS-08-1060P2 and MDS-14-1611).

### Treatment: environmental enrichment housing conditions and (-)-epigallocatechin-3-gallate (EGCG)

Ts65Dn and WT 5–6 months old female mice were randomly assigned to one of the following experimental groups: no treatment (NT), environmental enrichment (EE), green tea extract containing 45% (-)-epigallocatechin-3-gallate (EGCG), or a combination of EE and EGCG (EE-EGCG). Mice received the different treatments for 30 days based on previous studies (Pons-Espinal et al., 2013; De la Torre et al., 2014). In the NT condition animals were reared in conventional cages (20 × 12 × 12 cm height, Plexiglas cage) in groups of 2–3 animals. EE housing consisted of spacious (55 × 80 × 50 cm height) Plexiglas cages with toys, small houses, tunnels, and platforms of different shapes, sizes, colors and textures. Wheels were not introduced in the cages in order to avoid the effect of physical exercise. The arrangement was changed every 2 days to keep novelty conditions. To stimulate social interactions, 6–8 mice were housed in each cage. EGCG was administered in drinking water (EGCG dosage: 0.326 mg/ml, 0.9 mg per day; 30 mg/Kg per day) by preparing fresh EGCG solution every 2 days from a green tea leaf extract [Mega Green Tea Extract, Decaffeinated, Life Extension®, USA; EGCG content of 326.25 mg per capsule]. Even if there were fluctuations in EGCG dosage due to drinking volume and mice weight there were no significant differences in mean EGCG intake along days between WT (29.79 mg/Kg per day) and Ts65Dn (32.59 mg/Kg per day) mice (data not shown). The sample size for each experimental group was the following: WT = 10; TS = 11; WT-EE = 14; TS-EE = 11; WT-EGCG = 11; TS-EGCG = 9; WT-EE-EGCG = 12; TS-EE-EGCG = 8.

### Morris water maze

The water maze consisted of a circular pool (1.70 m diameter; 0.6 m height) filled with tepid water (19 ± 2°C) opacified by the addition of white non-toxic paint. White curtains with affixed black patterns surrounded the maze to provide an arrangement of spatial cues. The settings enabled a spatial allocentric learning and memory task based on distal cues (Vorhees and Williams, 2006). The first day mice were habituated to the task at the pre-training session in which the escape platform (12 cm diameter, height 24 cm) was located at the center of the pool and was visible by 1 cm over the water level. During the following 5 days mice learned the position of the platform, which was hidden 1 cm below water (northeast quadrant, 22 cm away from the wall) in 4 training (acquisition) trials per day. In each trial, mice were placed at one of the starting locations in random order (north, south, east, west), including permutations of the four starting points per session, and were allowed to swim until they located the platform. Mice failing to find the platform within 60 s were placed on it for 20 s and were returned to their home cage at the end of every trial. To assess the reference memory a probe session was performed 24 h after the last acquisition session. The platform was removed and mice were allowed to swim for 60 s during which the % of time spent in the target quadrant and the proximity to platform (Gallagher index) was calculated by sampling the position of the animal in the maze (10 times per second) to provide a record of its distance to the escape platform in 1-s averages (Gallagher et al., 1993). The cued session was performed to test the mice motivation to find the platform and visual ability using the platform elevated 1 cm above the water with its position clearly indicated by a visible cue (black flag). Mice that did not reach the platform in less than 30 s in this session were considered unsuitable for the test and were subtracted from the analysis. During days 8–10, cognitive flexibility, the ability of mice to re-learn a new location of the platform, was assessed in the reversal sessions in which the platform was located at the opposite quadrant. There was 1 missing subject on the reversal sessions.

All the trials were recorded with an image tracking system (SMART, Panlab, Spain) connected to a video camera placed above the pool. Escape latencies, length of the swimming trajectories and swimming speed for each animal and trial were monitored and computed. The analysis of mice performance was conducted using a custom-designed analysis program, Jtracks software, which generates heat-maps of the spatial distribution of the accumulated trajectories in each group. Jtracks was further used to obtain other measurements such as the Gallagher index and the Whishaw index, defined as the percentage of path inside the optimal corridor connecting release site and goal, to quantify the most efficient and direct trajectory from the location of mice to the platform (Whishaw and Jarrard, 1996).

### Statistical analysis

Two questions were addressed: the global differences over time and the progression of learning across sessions. The first question was tested by single variate analysis of the differences between experimental groups for three learning-related parameters (latency to reach the platform, Gallagher index and % of time spent in the periphery). Data were expressed as mean + S.E.M and analyzed using One-way ANOVA or ANOVA repeated measures. The second question was evaluated by estimating the linear effect of time-group interaction using a general linear-mixed model for each behavioral parameter. We associated random-effects terms with the animal factor in order to model within-subject correlation that appears due to the repeated nature of the data. Also, the variable “latency” was right-censored, since mice are allowed to swim a maximum of 60 s (Vock et al., 2012). Estimation of the coefficients and their associated *p*-values were based on maximum log-likelihood methods using the R library censReg (Henningsen, 2013). We used the plot of the model residuals vs. the fitted values to check model assumptions. Multiple comparisons for parametric model were used to address *post-hoc* comparisons using multest R package and glht function (Hand and Taylor, 1987; Dickhaus, 2012). Non-treated WT and Ts65Dn were considered as the reference groups for the comparisons. To control the false discovery rate (FDR) due to multiple *post-hoc* comparisons Benjamini-Hochberg method was used (Benjamini and Hochberg, 1995). This procedure was implemented both for the ANOVA and for the linear-mixed model in the R package multtest (Pollard et al., 2005).

### Principal component analysis

The “learning” process is composed by many variables whose influence on performance may be great for some, whereas for others it may be so small that they can be ignored. For example, you might start with ten original variables, but might end with only two or three meaningful axes. This is known as reducing the dimensionality of a data set. PCA is the most commonly used technique to identify linear combinations of variables in a high-dimensional space best representing the variance that is present in the data. This is achieved by considering each variable to be an axis in a high-dimensional space. Individuals, or groups of individuals, can be represented as points in this space. PCA identifies a linear combination of the original variables, called principal component that accounts for the largest amount of the experimental variability. Once this first principal component is set, PCA finds successive orthogonal principal components that explain the maximum amount of the remaining variance given that the orthogonality constraint is met. Finally, the original data and the original variables can be projected in this new space defined by the principal components. In our analysis we were mainly interested in the variation among experimental groups as well as the variation of a given group along the learning sessions. To find the variables best representing these two types of between-group variation (within- and between-learning sessions), we used the group medians of each variable on each acquisition day. A supervised analysis using group means instead of variables measured on individuals is known as discriminant analysis, (c.f. Greenacre, 2010). Such methods are suitable for the analysis of behavioral data having several conditions with a number of replicates per condition. For reasons of robustness to outliers, however, we here prefer to use the medians instead of the means. The PCA was performed on 40 observations (eight experimental groups on five learning sessions, where the four trials of each learning session were averaged) corresponding to median group performances of seven variables on each acquisition day. Separately, a similar analysis was done for the three reversal sessions.

The variables of interest were latency to target, percentage of time spent in target quadrant, percentage of time spent in the periphery, Whishaw index, Gallagher index, distance traveled, and speed. To allow for the combination of the original variables measured in different units, all variables were scaled to unit variance before the analysis (the default *Z*-score scaling was used).

Since the PCA was performed on group medians (grouped data), points identified in the PCA space will correspond to groups of individuals. To identify points corresponding to individuals themselves, we used the technique of “adding supplementary points.” Given a single measurement corresponding to a point in the space of the original variables, we can identify the new coordinates of this point in the space defined by the principal components. Note that such points will not change the coordinate system, as they are added after the PCA is performed. Adding all 86 individuals appearing five times each as supplementary points, we identified the coordinates for each of the individuals. The R-package FactoMineR (Lê et al., 2008) was used for the PCA as it allowed for a straightforward inclusion of supplementary observations. Density plots were obtained using the statdensity_2d function from the ggplot2 R package (Wickham, 2009) with the parameters: *n* = 100, *h* = 5, and bins = 6.

### Permutation test

To assess statistical significance of group separation, we performed a permutation test, a standard procedure in multivariate data analysis (Sham and Purcell, 2014). Individuals were drawn and reassigned randomly to experimental groups. Correct acquisition sessions were maintained, and thus each individual kept their learning performance along acquisition (i.e., all five values corresponding to the learning sessions of an individual were assigned to the same group). Group medians were then determined for each learning session for these new groups. Original numbers of individuals in each group were kept. To determine overall group separation, percentage of within-session variance (see variance decomposition below) was used as a statistic. For learning differences, we used a *t*-statistic involving PC1 pairwise group comparisons. All pairwise comparisons were determined at each permutation. The number of randomized PCAs was 10,000.

### Variance decomposition

Total, between-group, between-session, and within-session variances were directly calculated from the (standard) coordinates obtained from the PCA. Variance in the PCA was calculated from the distances *d* of objects *i* from the origin:

$$\begin{array}{l} V=\frac{1}{7N}\overset{N}{\underset{i=1}{\sum }}d\begin{array}{c} 2\\ i \end{array} \end{array}$$

where the factor 1/7 comes from the number of variables. In the case of between-group variance *V*_*B*_, the objects *i* are the groups and *N* = 40. Since we performed the PCA on the groups, by construction the between group variance sums to 1. For the total variance *V*_*T*_, the objects are the individuals (supplementary points) and *N* = 430. The percentage of between-group variance is then *V*_*B*_/*V*_*T*_ x 100. The usual definition of *V*_*B*_ is for the group averages, not medians, which means that here we are actually estimating a lower bound on the percentage of between-group variance. To obtain the between-sessions variance *V*_*BS*_, we calculated the squared mean distance from the origin over all groups on a given acquisition session *s*:

$$\begin{array}{l} d\begin{array}{c} 2\\ s \end{array}=\overset{7}{\underset{p=1}{\sum }}{\left(\frac{1}{8}\overset{8}{\underset{j=1}{\sum }}x_{s,p,j}\right)}^{2} \end{array}$$

where the *x*_*s, p, j*_ are the standard coordinates for principal axis *p* of an experimental group *j* during a given session *s*. Then we used the first formula for the variance with *N* = 5. Within-session variance is the difference *V*_*B*_ – *V*_*BS*_ (which can again be expressed as a percentage of total variance *V*_*T*_).

## Results

To evaluate the functional impact of EE, EGCG, and the potential synergistic effects of a combination of EE-EGCG on the age-associated hippocampal-dependent learning and memory deficits of Ts65Dn mice, we compared the behavioral performance of mice treated with EE, EGCG, or EE-EGCG in the MWM with their untreated controls of both WT and Ts65Dn genotypes.

Two different questions were addressed: the overall learning differences among the experimental groups on single learning variables (escape latency, Gallagher index etc.) and the effects of treatments on the progression of learning across sessions (slope of the learning curve). The first question was evaluated by analyzing the effect of the group variable (defined by genotype and treatment) by one-way repeated measures ANOVA. The second question was evaluated by estimating the linear effect of time-group interaction with a general linear-mixed model on each behavioral parameter, censored for latency.

### Effects of EE, EGCG, and EE-EGCG treatments

**Figure 1:**
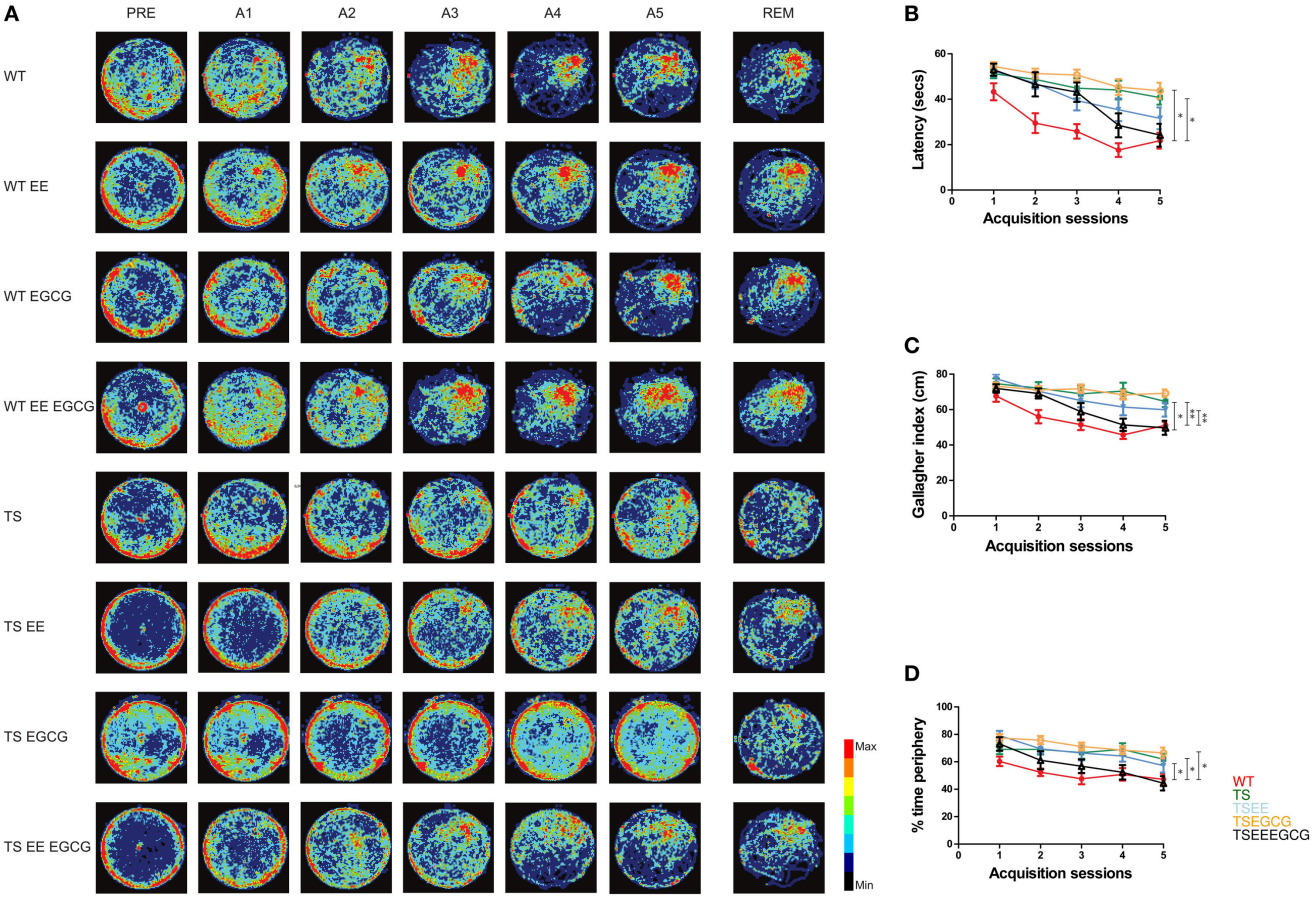
**EE-EGCG treatment is more efficient than EE or EGCG alone to ameliorate the hippocampal-dependent learning and memory alterations of middle age Ts65Dn mice**. **(A)** Heat-map representing the accumulated trajectories of mice from the different experimental groups [untreated wild type (WT) = 10, untreated Ts65Dn (TS) = 11 WT-EE = 14; TS-EE = 11; WT-EGCG = 11; TS-EGCG = 9; WT EE-EGCG = 12; TS EE-EGCG = 8] across sessions in the Morris water maze. Color scale is depicted on the right, where red corresponds to the most visited zones and black to the less or non-visited zones. **(B)** Latency (seconds to reach the escape platform). **(C)** Gallagher index (mean distance between subject and goal in cm). **(D)** Thigmotaxis (percentage of time spent on the periphery). Data in **(B–D)** are represented as mean ± SEM. Data were analyzed with ANOVA repeated measures with Tukey *post-hoc* comparisons corrected with BH; ^*^p < 0.05, ^**^p < 0.01. PRE, pre-training; A1-5, acquisition days 1–5 with 4 trials per day; REM, removal. All the possible *post-hoc* comparisons were performed but only treated TS, and untreated WT and TS groups are shown in the figure.

First we performed a classical single-variate analysis including relevant parameters for the learning process. During the habituation (pre-training) session, all groups behaved in a similar manner, with no differences in the latency to reach the visible platform [overall genotype-treatment effect *F*_(7, 78)_ = 0.937; *p*-value *n.s.*] or the mean distance to the platform, as quantified by the Gallagher index [overall genotype-treatment effect *F*_(7, 78)_ = 1.161; *p*-value *n.s.*] indicating no genotype- or treatment-dependent differences in procedural learning (Figure 1A).

**Figure 2:**
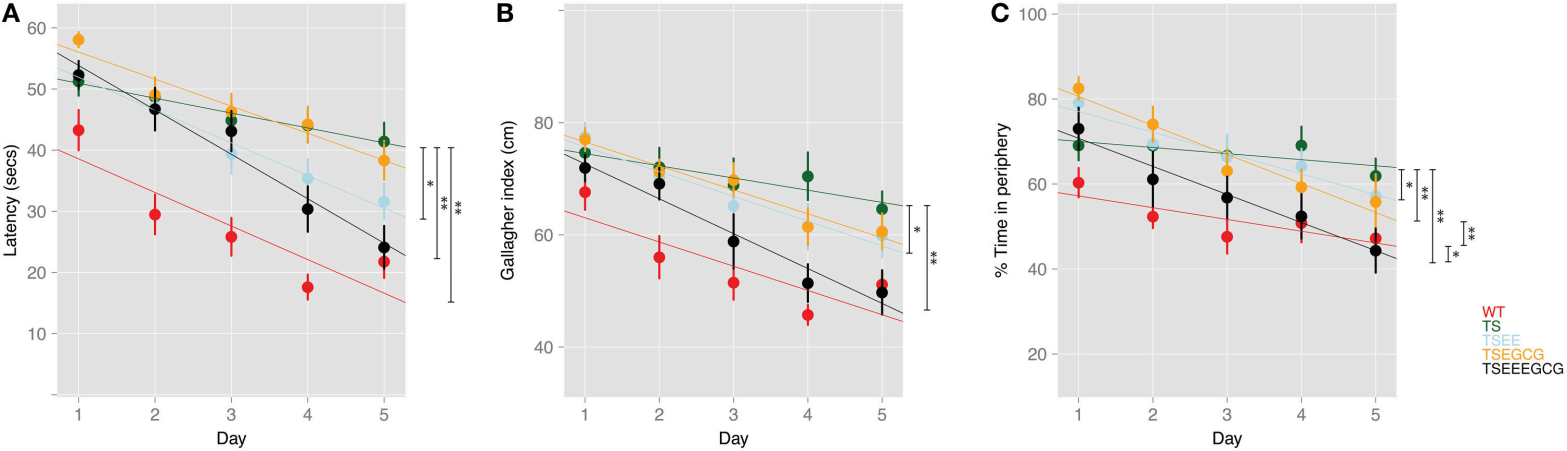
**Linear mixed model reveals improvement of hippocampal-dependent learning upon EE and EE-EGCG treatment in middle age Ts65Dn mice**. Fitted linear mixed model (represented as colored lines) and observations (dots) represented as mean ± SEM of **(A)** Latency to reach the escape platform (log-transformed censored model), **(B)** Gallagher index, and **(C)** thigmotaxis along learning sessions. The model enabled the comparison of the slope of the learning trajectory (β) across days among experimental groups. *Post-hoc* comparisons were corrected with BH; ^*^p < 0.05, ^**^p < 0.01; non-treated WT and TS were considered as references for the comparisons.

Along the acquisition sessions, untreated WT mice efficiently learned the platform position, as shown by the progressive reduction in the latency to reach the hidden platform and the increasing preference for the target quadrant (Figures 1A–C). As it has been previously reported, we detected impaired learning ability in untreated Ts65Dn mice, shown by the higher latency to reach the hidden platform across days that was not reduced across sessions, leading to a flatter learning curve (β = −3.05; *p*-value < 0.01, Figure 2A) in comparison to untreated WT. Trisomic mice also showed increased global Gallagher index [overall genotype-treatment effect *F*_(7, 78)_ = 7.072, *p*-value < 0.01; Tukey *post-hoc* BH corrected *p*-value < 0.01, Figure 1C] and the typical increased thigmotaxis [higher percentage of time spent close to the pool periphery; overall genotype-treatment effect *F*_(7, 78)_ = 6.12, *p*-value < 0.01; Tukey *post-hoc* BH corrected *p*-value < 0.05, Figure 1D] that has been previously reported (Reeves et al., 1995).

EE ameliorated the deficits found in Ts65Dn mice as shown by a reduction of the escape latency across acquisition sessions as compared to untreated trisomic mice (β = −2.92; *p*-value < 0.05, Figure 2A). Interestingly, enriched Ts65Dn (TS-EE) mice also exhibited a more goal-directed behavior as shown by a progressive reduction of the Gallagher index (β = −2.23; *p*-value < 0.05, Figure 2B) and of thigmotactic behavior (β = −3.47; *p*-value < 0.05, Figures 1A, 2C) in comparison to untreated trisomic mice. However, Ts65Dn mice under EE still showed poorer performance when compared to WT as reflected by higher Gallagher index values (Tukey *post-hoc* BH corrected *p*-value < 0.01, Figure 1C) and thigmotaxis (Tukey *post-hoc* BH corrected *p*-value < 0.05, Figure 1D).

Conversely, Ts65Dn mice treated with EGCG (TS-EGCG) did not show any effects of treatment. In this group, neither the latency to reach the platform (β = −1.99; *p*-value = *n.s.*, Figure 2A) nor the Gallagher index (β = −2.09; *p*-value = *n.s.*, Figure 2B), were improved as compared to untreated Ts65Dn mice. In fact, TS-EGCG mice exhibited increased thigmotactic behavior (β = −5.39; *p*-value < 0.01, Figure 2C).

Finally, the combined treatment with EE-EGCG significantly improved performance in Ts65Dn mice, markedly reducing the latency to reach the platform (β = −4.83; *p*-value < 0.01, Figure 2A), Gallagher index (β = −4.04; *p*-value < 0.01, Figure 2B) and thigmotaxis (β = −5.18; *p*-value < 0.01, Figure 2C) across the acquisition sessions as compared to untreated trisomic mice. In fact, the combined treatment effects in Ts65Dn mice reached values that were not statistically different from those of untreated WT mice in latency (β = −1.78; *p*-value = *n.s.*, Figure 2A), Gallagher index (Tukey *post-hoc* BH corrected *p*-value = *n.s.*, Figure 1C) and thigmotaxis (Tukey *post-hoc* BH corrected *p*-value *n.s.*, Figure 1D) suggesting a rescue of the phenotype.

Neither of the treatments had effects on the latency to reach the platform, nor on the Gallagher index in WT mice. However, both EE (β = −3.11; *p*-value < 0.05, Figure S1) and EE-EGCG (β = −3.94; *p*-value < 0.01, Figure S1) promoted a significant reduction in the percentage of time in the periphery along acquisition days.

There were no differences in swimming speed between untreated WT and Ts65Dn mice (overall gen-treatment effect *F*_(7, 8)_ = 2.820; *p*-value < 0.05; Tukey *post-hoc* comparisons corrected by BH showed *p*-value = *n.s.*; data not shown). On the other hand EGCG treatment had a significant effect reducing swimming speed on WT (Tukey *post-hoc* BH corrected *p*-value < 0.05; data not shown) and Ts65Dn (Tukey *post-hoc* BH corrected *p*-value < 0.01; data not shown) in comparison with untreated WT. The rest of the treatments showed no effect on swimming speed during learning.

### Effects of EE, EGCG, and EE-EGCG treatments on reference memory and cognitive flexibility

**Figure 3:**
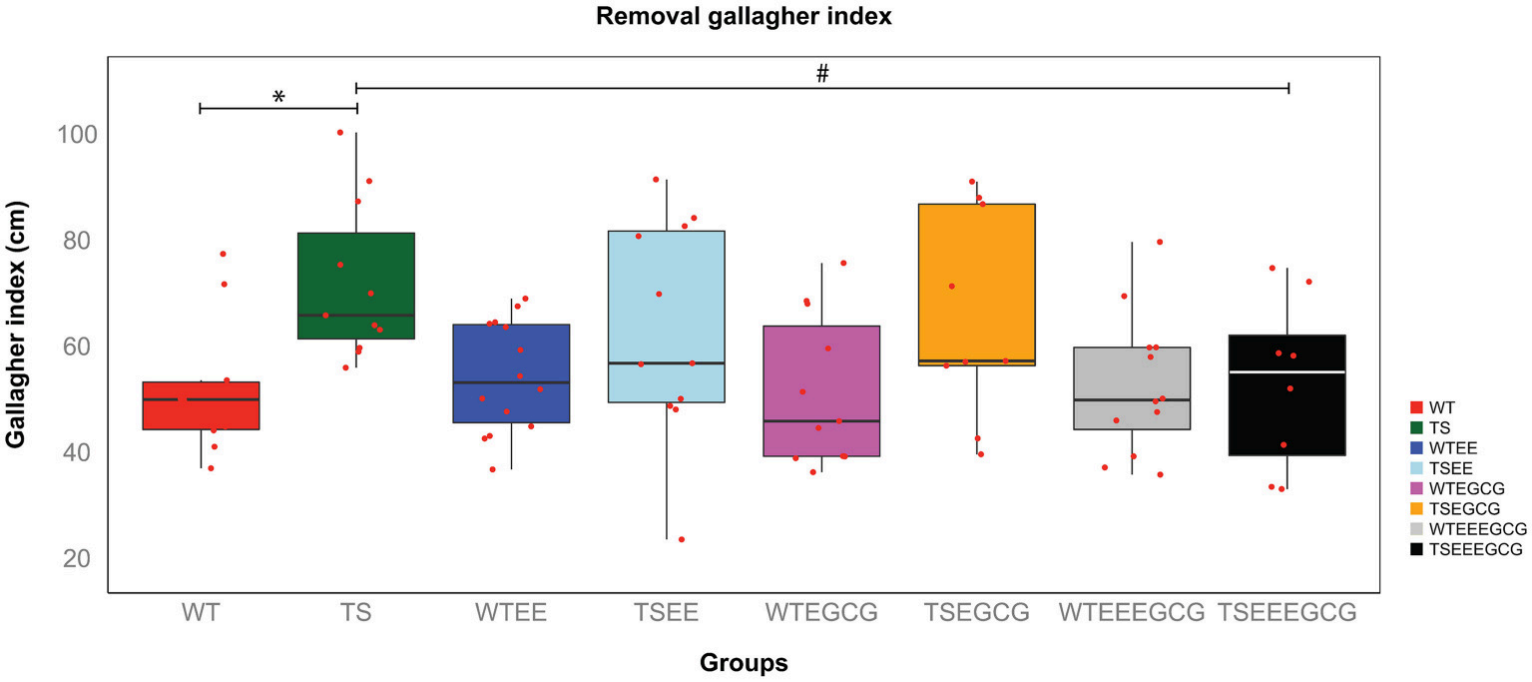
**EE-EGCG treatment is more efficient than EE or EGCG alone to ameliorate reference memory at the probe trial in Ts65Dn group**. The figure shows boxplots of the distribution of the distance to the target (Gallagher index) of all experimental groups in the removal session. In each boxplot, the horizontal line corresponds to group median, the box edges gives the 25th and 75th percentiles and the whiskers depict minimum and maximum values to a maximum of 1.5 times the interquartile distance from the box, and more extreme values are individually plotted. Red dots indicate the values of each individual mouse. TS-EE-EGCG showed a reduction of Gallagher index when compared to untreated TS mice that is not observed in the rest of TS mice groups. ANOVA, with Tukey *post-hoc* comparisons corrected with BH; ^*^p < 0.05; ^#^*p* = 0.05; comparisons were performed to test the differences between genotypes and the effects of treatments on the TS.

To assess the reference memory a probe trial was performed 24 h after the last acquisition day. The percentage of time spent in the target quadrant showed a tendency in untreated Ts65Dn to perform worse than WT and also in EE-EGCG treated Ts65Dn mice to perform better than untreated Ts65Dn, however there were no statistically significant differences among the groups [overall genotype-treatment effect *F*_(7, 78)_ = 1.498; *p*-value = *n.s.*; Figure S2]. This was probably due to the large within-group variance as depicted in the boxplots, by the large distance between the box edges (25th and 75th percentiles; Figure S2). On the other hand, the Gallagher index, which is a more precise measure, presented less within-group variance and showed global differences in performance among experimental groups [overall genotype-treatment effect *F*_(7, 78)_ = 2.741; *p*-value < 0.05, Figure 3]. Tukey *post-hoc* comparisons adjusted by the BH method showed that untreated Ts65Dn presented higher Gallagher index than untreated WT mice (*p*-value < 0.05) indicating poor reference memory. The administration of EGCG (Tukey *post-hoc* BH corrected *p*-value = *n.s*) or EE (Tukey *post-hoc* BH corrected *p*-value = *n.s*) alone did not affect the Ts65Dn reference memory deficit. However, the combination of EE-EGCG reduced the Gallagher index in Ts65Dn (Tukey *post-hoc* BH corrected, *p*-value = 0.05), reaching a performance that was similar to WT (Tukey *post-hoc* BH corrected *p*-value = *n.s.*, Figure 3).

Cognitive flexibility was assessed along the reversal sessions. While untreated WT mice clearly shifted their search to the new platform location, untreated Ts65Dn mice persevered searching the old platform location. This poorer cognitive flexibility was reflected in an increased latency to reach the new platform position [overall genotype-treatment effect *F*_(7, 77)_ = 5.648, *p*-value < 0.01; Tukey *post-hoc* BH corrected *p*-value < 0.01, Figure S3A], a trend toward an increased Gallagher index [overall genotype-treatment effect *F*_(7, 77)_ = 7.438; Tukey *post-hoc* BH corrected *p*-value = 0.06, Figure S3B] and increased thigmotaxis across the 3 reversal learning sessions [overall genotype-treatment effect *F*_(7, 77)_ = 4.570; Tukey *post-hoc* BH corrected *p*-value < 0.05; Figure S3C], as compared to WT. Even though there were no significant effects of any of the treatments on Ts65Dn latency to reach the new platform positions, both TS-EE (β = 11.78, *p*-value < 0.05, Figure S3A) and TS-EE-EGCG (β = 11.95, *p*-value < 0.05, Figure S3A) were qualitatively less different from WT than untreated Ts65Dn mice (β = 18.46, *p*-value < 0.01, Figure S3A) taking into account the magnitude of the group differences by the model estimate (β). Neither of the treatments had effects on the Gallagher index (Figure S3B) nor the thigmotaxis on Ts65Dn or WT mice during the reversal sessions (Figure S3C).

### Multidimensional analysis of learning impairment in Ts65Dn mice: global effects of EE, EGCG, and EE-EGCG treatments

There is not a single best measure of learning (such as the classical “escape latency” or “distance traveled”) and thus, discrimination of learning performance differences could be better achieved by a combination of some of these variables. Thus, to go a step further, we used permutation-validated principal component analysis (PCA), to determine which combination of the experimental variables would be best suited to describe the differences in learning among our groups. Significance of differences was determined by permutation-based test statistics.

**Figure 4:**
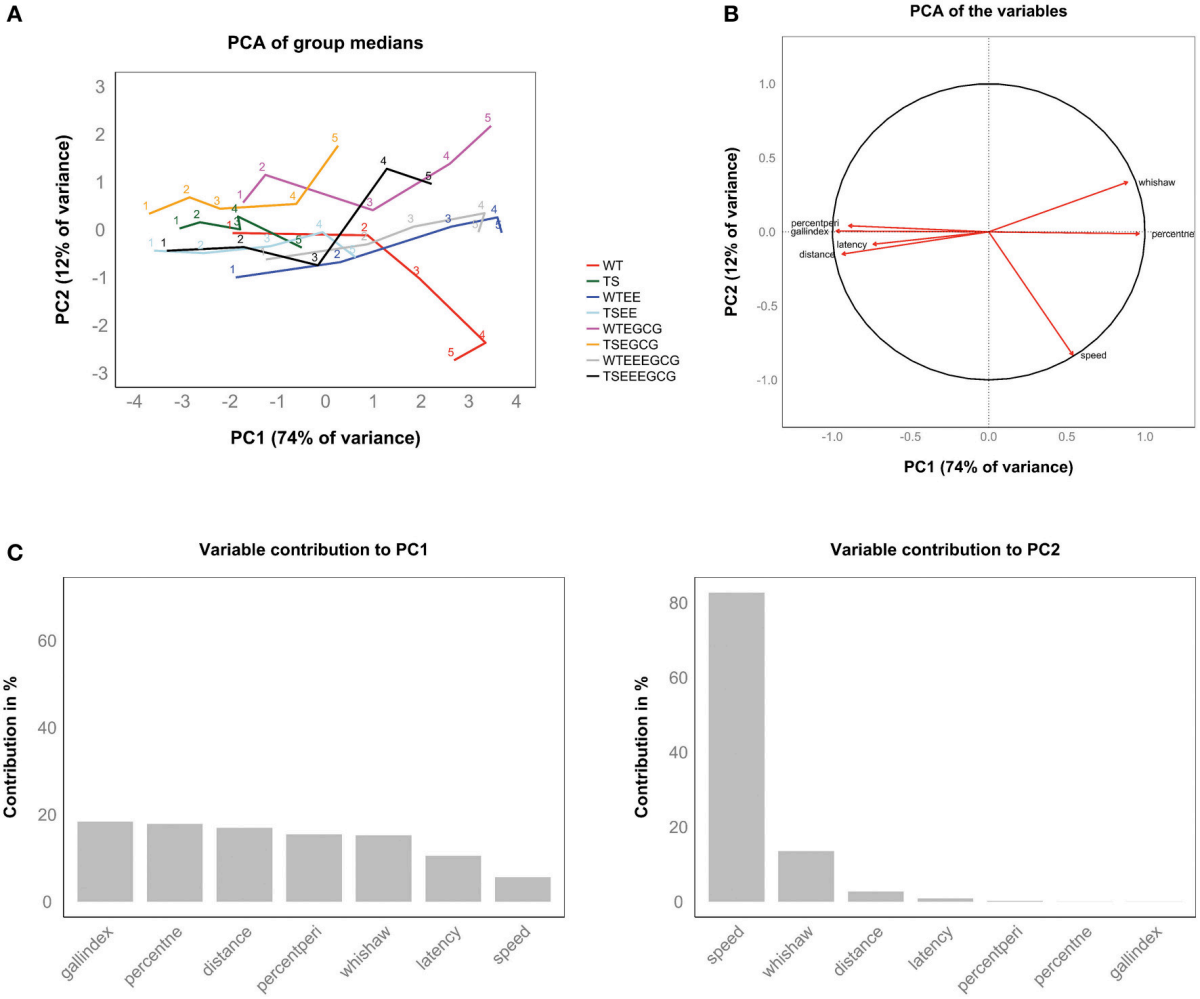
**Supervised PCA of the experimental groups during the acquisition sessions revealed the main direction of learning along the first principal component**. **(A)** Distribution of the group performance (medians) in the new ordination space, which consists of linear combinations of the original variable space. Each trajectory represents an experimental group and connects the five learning sessions labeled with its respective number. All group trajectories showed a progression toward positive values of the first principal component (PC1). For a given learning session, experimental groups achieving better performance attain higher values on this axis. The progression of trajectories on the second principal component (PC2) appears more erratic. **(B)** PCA of the variables, where arrows represent the direction of each variable in the PCA space. Arrows reaching the unit circle belong to variables that are well represented by the two principal components. **(C)** Bar plots showing the percentage of explained variance for each principal component. Bars represent the contribution (%) of each variable to first and second principal components. The first principal component (left panel) can be interpreted as a composite learning variable where classical variables used to assess learning had major and similar contribution ranging from 18% in the case of the Gallagher index to 10% in the case of the latency. Speed (right panel) constitutes the main contributor to PC2 (82%), but is split between PC1 and PC2 in almost equal parts (see panel **B**).

Variables related to the learning improvement along the five learning sessions, including Gallagher index, % time spent in target quadrant, distance traveled, percentage of time spent in periphery, Whishaw index and latency to target, loaded on PC1, which accounted for 74% of the (between-group) variance (Figures 4A,C). High values of PC1 correspond to short distances to target, low latencies, high percentages of time in the target quadrant, etc. (Figure 4B). This axis can be understood as a new composite learning measure. In contrast, the second principal axis (PC2, 12% of variance) is dominated by the contribution of swimming speed and thus is mainly dependent on motor ability. By construction, it is independent from the learning-related PC1. It is noteworthy that speed also contributes to PC1, where it shows a relation to learning (animals that have learned the target position tend to go there faster). Speed is thus decomposed in a learning-dependent component and a learning-independent component more related with the intrinsic motor capability of mice (Figure 4B).

Each of the eight experimental groups is represented as a trajectory connecting five dots that correspond to the five learning sessions (see Figure 4A). Each group trajectory shows a main direction from left to right (along PC1) that represents the group's overall learning and off-target speed (speed in swim paths not goal-directed). For instance, the untreated Ts65Dn group trajectory reaches a maximum value of PC1 at the end of the learning phase (last learning session corresponding to their best performance level) that corresponds to initial PC1 values (learning sessions 1 and 2) of the untreated WT trajectory, indicating poor learning associated with the trisomy. Interestingly, the Ts65Dn group treated with EE-EGCG shows a trajectory that advances well into the right quadrants, attaining maximum values of PC1 that equal those reached by untreated WT at the end of the learning phase (efficient learning trajectory). There are also interesting differences in the second dimension (PC2). The most striking is that untreated WT follow an opposite trajectory to the EGCG-treated WT. Both groups reach the lowest and highest values of PC2, respectively, indicating opposite changes in swimming speed during learning upon treatment. Generally, trajectories of EGCG-treated groups have higher values of PC2 than their untreated counterparts (with significant differences in PC2 between the EGCG treated WT and the untreated WT group, as well as between the EGCG treated Ts65Dn and the untreated Ts65Dn group on session 5, by permutation test). This indicates a general reduction in swimming speed due to EGCG treatment (data not shown).

To assess the statistical significance of these differences, we determined the amount of individual variation within each group by mapping the position of each individual on each acquisition day to the PCA plot (see Materials and Methods). As shown in Supplementary Figure 4, there is a substantial amount of individual variation across the learning sessions in all the experimental groups. In fact, the within-group variance attains 60% of the total variance (the between-group variance amounts to about 40% of the total, see Materials and Methods). Part of the between-group variance stems from the variance among learning sessions, so that the amount of between-group variance can further be decomposed into between-learning sessions (17%) and within learning-session (23%). While the former quantifies how an average group performance varies across learning sessions, the latter quantifies the average separation of the experimental groups. This separation is highly significant (*p* < 10^−4^, permutation test, see Materials and Methods).

**Figure 5:**
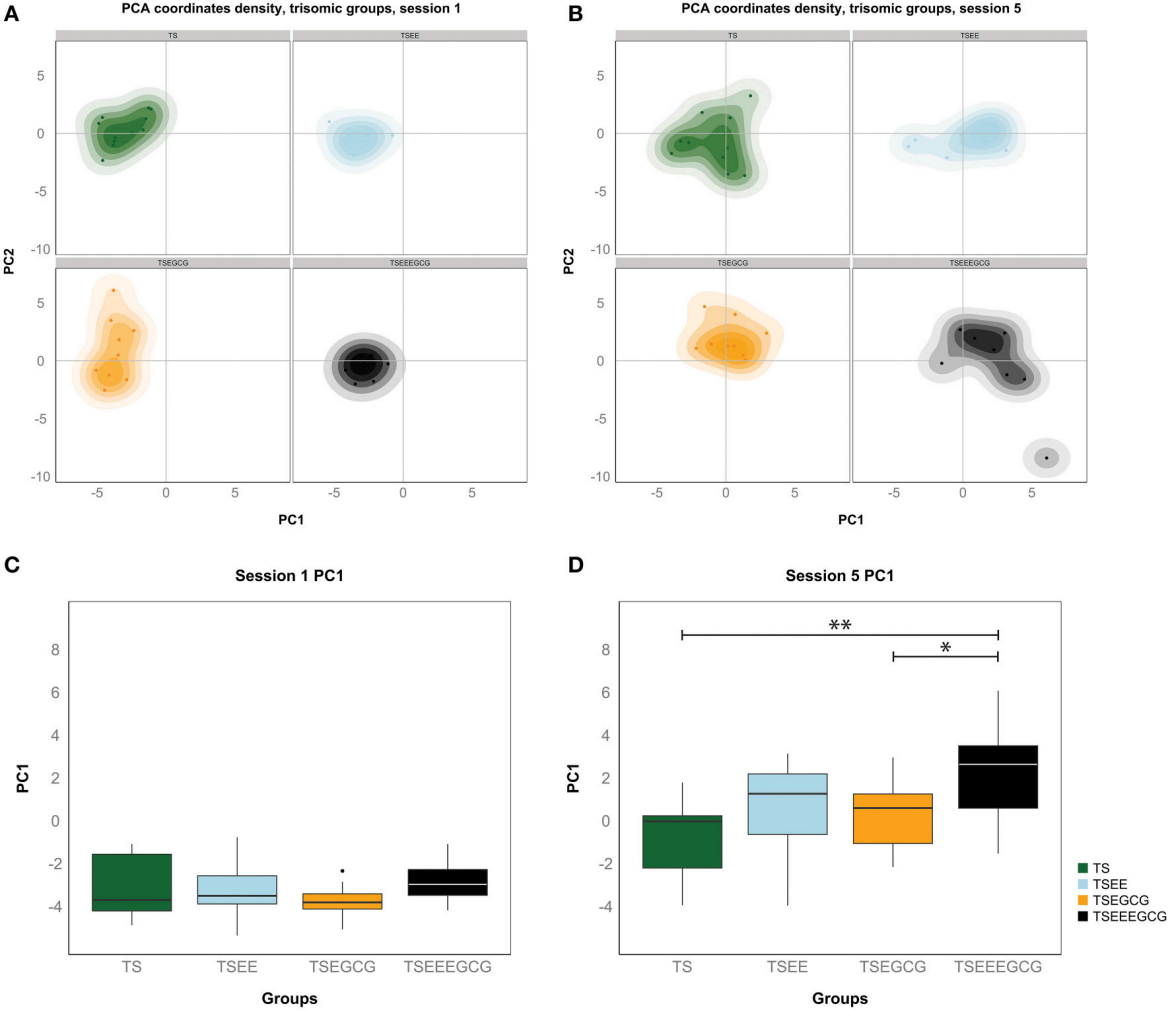
**The first principal component of the PCA (PC1) discriminates good learners from poor learners in the Ts65Dn groups**. **(A)** Density distribution of all Ts65Dn groups for the first and the second principal components of the PCA (PC1 and PC2). On the first acquisition session all Ts65Dn groups showed a similar value on PC1, which can be interpreted as a composite variable explaining learning, indicating a comparable basal performance of all Ts65Dn animals. **(B)** TS-EE and TS-EE-EGCG mice manifested higher values of PC1 on the fifth acquisition session, at the end of the learning phase, explained by the benefits of the treatment on the learning process on these groups. On this session groups were also more spread because within group individual phenotypic differences also increased during the learning process. Boxplots of the distribution of the first principal component for each Ts65Dn group on the first **(C)** and the fifth **(D)** session of the acquisition phase. In each boxplot, the horizontal line corresponds to group median, the box edges gives the 25th and 75th percentiles and the whiskers depict minimum and maximum values to a maximum of 1.5 times the interquartile distance from the box. More extreme values are individually plotted. TS-EE-EGCG reached significant higher values on the composite learning variable than TS and TS-EGCG mice on the fifth acquisition session. Permutation test ^*^p < 0.05, ^**^p < 0.01.

Figure 5 density plots show the individual variation within the Ts65Dn experimental groups during the learning process. While individuals started off from similar positions on learning session 1 (Figure 5A), on session 5 (Figure 5B) the trisomic groups spread out indicating increasing variation along the learning process. This would represent the phenotypic variability that is specifically due to learning, whereas the variation in baseline (contributed by other motivational or motor factors) is much smaller. Statistical significance of differences in learning was evaluated via a permutation test involving a *t*-statistic based on PC1. This analysis showed significant differences between the EE-EGCG treated and untreated Ts65Dn (*p*-value < 0.01) and EE-EGCG treated and EGCG treated Ts65Dn (*p*-value < 0.05) at the end of the learning period (session 5, Figure 5D), which were not observable during the first learning session (Figure 5C). WT mice manifested a more homogenous behavior with all groups starting from similar values in session 1 (Figure S5A) and reaching similar learning performance in session 5 (Figure S5B). WT mice showed no significant difference among treatments neither in the first session (Figure S5C) nor at the end of the learning process (Figure S5D). Significant pairwise group comparisons during learning sessions 1 and 5 based on PC1 can be found in Supplementary Tables 1, 2, respectively.

We used the same approach to analyze the data from the reversal sessions. In this case, PC1 can be interpreted as a learning composite variable explaining cognitive flexibility (Figure S6). We observed that both Ts65Dn (Figures S7A, S7B) and WT (data not shown) mice achieved higher values on PC1 along the sessions. However, there were no significant effects of the treatments within the same genotype on the last reversal session (Figure S7D), although there is a trend toward higher values of PC1 for the EE-EGCG Ts65Dn group which almost reaches significance (*p*-value = 0.08, Figure S7D). No significance differences were detected on the first reversal session (Figure S7C). It is likely that a greater number of sessions would increase the difference between double treated trisomics and untreated ones in a similar way as in the acquisition. Significant pairwise group comparisons during reversal sessions 1 and 3 based on PC1 can be found in Supplementary Tables 3, 4, respectively.

## Discussion

Individuals with DS undergo a progressive age-associated neurodegenerative process that resembles that of AD. Early signs of dementia in people with DS are the dysfunction of the frontal lobe and hippocampus, where amyloid first accumulates during the early stages. Cognitive symptoms of dementia in people with DS are similar to those of AD patients and include forgetfulness, impaired short-term memory, confusion, learning problems, and deficits in visuospatial organization (Lott and Dierssen, 2010). Some of these symptoms are recapitulated in DS mouse models, such as the Ts65Dn mice (Holtzman et al., 1996; Granholm et al., 2000).

The present study was aimed to investigate the potential of a combined treatment with EE and a green tea extract containing EGCG to ameliorate the hippocampal-dependent spatial learning and memory deficits in Ts65Dn mice at the age of the onset of cognitive decline. Besides the classical single-variate analysis, we applied here a novel multidimensional approach for the analysis of the effects of the different genotypes and treatments. To achieve the best discrimination between groups we used a supervised PCA involving the group medians on each acquisition session of a number of behavioral variables that are differentially modified during the learning process (see Materials and Methods). PCA has been applied to MWM analysis before. In a study from Keeley and McDonald (2015) a number of MWM navigation-related variables are mixed with variables characterizing the individuals to then identify the main contributors to overall variance as obtained by PCA. A very comprehensive MWM-related PCA study (Wolfer and Lipp, 2000) analyzed over 3000 mice from a large number of individual experiments. The approach allowed identifying a large degree of variance unrelated to spatial learning and was used to warn about oversimplified approaches disregarding variation caused by memory-unrelated effects. Our approach is rather different since it works on the group level and analyzes separately the different types of sessions. The amount of variance unrelated to genotype or treatment is taken into account by evaluating the within-group variance separately (which also enables a permutation-based significance analysis). This approach is known as discriminant analysis, and in its linear variant (LDA) has been used to classify swim paths in the MWM (Graziano et al., 2003). Our discriminant analysis based on PCA allowed depicting the 5-day trajectories of each experimental group through a space spanned by a speed-related variable and a composite learning variable. This composite learning variable reflects global treatment-induced learning differences. Traditionally, PCA does not address exact hypothesis testing, and is only used to identify which variables account for large proportions of variance in data sets, which can then inform the choice of statistical tests among variables for ANOVA testing. Here, we applied a permutation test procedure that allowed for precise statistical significance estimations both in terms of explained variance and with respect to distances in PC1. This test has the advantage that it does not require a *post-hoc* correction for multiple comparisons or many variables. PC1 combines similarly sized contributions from six main learning-related variables that accounted for 74% of the between-group variance along the five learning sessions, and all variables, except speed, load similarly on this composite learning performance measure. This argues that all these variables capture the same amount of information concerning learning, and although in some aspects they may be redundant, they are essentially measuring slightly different learning aspects. In contrast, the second principal axis (PC2, 12% of variance) is dominated by the contribution of swimming speed and thus is mainly dependent on motor ability. However, speed also contributes to PC1, and is thus decomposed into a learning-dependent component (mice go faster to a target they have learned) and a learning-independent component (related with intrinsic motor capability). We also applied our multidimensional analysis to the reversal sessions. In this case, PC1 can also be interpreted as a composite variable explaining learning (re-learning of a new platform location related to cognitive flexibility).

As previously described, in our study both single-variate and PCA analysis showed spatial learning impairment in 6–7 months old Ts65Dn mice, as reflected along the acquisition sessions by an increased latency to reach the platform accompanied by increased Gallagher index and thigmotactic behavior as compared to WT mice. Ts65Dn performance reached a maximum value of PC1 (composite learning measure) in the last learning session, which corresponded to initial PC1 values (learning sessions 1 and 2) of untreated WT, indicating a global learning impairment in trisomic mice. In addition, Ts65Dn mice showed an impoverished reference memory, as indicated by the significantly increased Gallagher index in the probe trial (removal session). Finally, cognitive flexibility impairment was detected in the reversal sessions as revealed by increased permanence in the previously trained quadrant, which prevented an adequate search shift to the new location of the platform. These results confirm previous studies showing that performance of Ts65Dn mice in the MWM is indicative of poor learning strategies and hippocampal-dependent learning and memory dysfunction (reviewed by Dierssen, 2012). Such impairment is detected from early stages and undergoes an age-related decline due to degenerative processes in the septo-hippocampal system (Holtzman et al., 1996; Granholm et al., 2000). Interestingly, the density plots especially of the trisomic groups on PC1 and PC2 revealed an increased within-group variance after learning (Figure 5), suggesting that some trisomic individuals learned better than others.

Consistently with previous findings (Martínez-Cué et al., 2002; Dierssen et al., 2003; Baamonde et al., 2011; Chakrabarti et al., 2011) we found that 1 month exposure to an enriched environment (EE) had a moderate effect on spatial learning impairment of 5–6 months old female trisomic mice, improving the efficiency in learning strategies across the acquisition sessions as shown by a reduction in the Gallagher index, in thigmotaxis and in the latency to reach the platform. These results are consistent with previous findings showing that EE induces positive though limited behavioral effects in young Ts65Dn mice. In fact, in our experiments EE had no effects on the latency to reach the platform or on the Gallagher index in WT mice, although it promoted a significant reduction in their percentage of time in the periphery along acquisition days (Figure S1), suggesting a more exploratory behavior. This is consistent with previous work in this strain (Martínez-Cué et al., 2002) that also reported reduced distances traveled in the periphery in young WT females. Different factors could account for these moderate effects of EE including gender, genetic background, or age of initiation of EE exposure.

In our study, EGCG administered for 1 month at the age of 5–6 months, did not improve spatial learning of neither WT nor Ts65Dn mice, despite the promising previous results in young Ts65Dn (De la Torre et al., 2014). Furthermore, EGCG-treated Ts65Dn group showed a trajectory in the PCA which had higher values of PC2 than untreated Ts65Dn indicating a general reduction in swimming speed during learning upon treatment. Since at 5–6 months of age Ts65Dn mice already show some age-associated cognitive decline and AD-like neuropathology (Granholm et al., 2000), it could be speculated that 1 month of treatment with EGCG at the dosage used in this study is not sufficient to reverse these effects, even though we cannot discard that a chronic treatment, initiating the administration of EGCG at earlier ages, or increasing the dosage, could restore the cognitive deficits in older trisomic animals.

Interestingly, the administration of EGCG in combination with EE was the most efficient in improving the spatial learning and memory impairment in Ts65Dn mice. EE-EGCG treatment markedly reduced escape latency, Gallagher index, and thigmotaxis. The PCA showed that EE-EGCG treated Ts65Dn group had higher values of PC1 than the rest of Ts65Dn mice groups, attaining maximum values of PC1 that were equal to those reached by untreated WT at the end of the learning phase (efficient-learning trajectory), thus suggesting a recovery of the phenotype. Regarding the reversal learning, mainly dependent on the prefrontal cortex functional integrity (De Bruin et al., 1994), according to the single-variate analysis none of the treatments were able to counteract Ts65Dn deficits. The PCA showed that EE-EGCG treatment in Ts65Dn mice was able to induce a marginal effect (*p*-value 0.08) at the 3rd session. The differences in EE-EGCG effects during the acquisition and the reversal sessions may be due to dysfunctions in different neural systems affecting Ts65Dn mice, involving both the hippocampus and the prefrontal cortex, which may not be equally ameliorated by the treatments.

The fact that both EE and EE-EGCG promoted similar effects, suggests that the combined administration of EE-EGCG enhanced the beneficial effect of EE. In fact, many of the effects reported for EE are overlapping those reported upon EGCG treatment, such as neuroplasticity enhancement, antioxidant activity, anti-inflammatory function, neuroprotection, promotion of the non-amyloidogenic proteolytic pathway of APP and modulation of the kinase activity of DYRK1A (for a review see Xicota et al., 2015). Specifically, EE induces a reduction of Aβ plaques (Jankowsky et al., 2005; Lazarov et al., 2005; Berardi et al., 2007; Li et al., 2013; Polito et al., 2014), of oxidative stress (Mármol et al., 2015) and increase in neurotrophins such as NGF and BDNF at the basal forebrain and other brain regions affected both in AD and DS (Ickes et al., 2000; Birch et al., 2013). Additionally, in young Ts65Dn mice short- and long-term exposure to EE has shown to reduce inhibitory neurotransmission (Begenisic et al., 2011) and rescue hippocampal cell proliferation and neurogenesis within the dentate gyrus (Chakrabarti et al., 2011). A recent paper by Gundimeda et al. (2014) shed light on other possible mechanisms as they showed that EGCG was able to potentiate the neuritogenic ability of BDNF in PC12 cells which ectopically expressed TrkB, the BDNF high affinity receptor, through the interaction with its high-affinity target 67-kDa laminin receptor (67LR), a non-integrin type cell-surface associated protein that is present in various regions of the brain. Thus, we could speculate that EGCG may enhance the beneficial effect of EE due to synergistic cellular and molecular effects between EE and EGCG since they share common functions.

High within-group variance as illustrated in density plots showed that some individuals learned better than others. In Ts65Dn mice all the treatments increased variability, indicating that some individuals are more sensitive than others to the effects of EE and the combined EE-EGCG treatment. On the other hand, a quantitative evaluation of individual variance revealed statistically significant differences of the composite learning variable between the EE-EGCG treated Ts65Dn compared with their untreated or EGCG-treated counterparts at the end of the learning process. This indicates that EE-EGCG treatment is able to globally modify the learning related behavior.

In conclusion, we demonstrated here that combined treatment of EGCG and EE had beneficial effects on age-related cognitive impairment in Ts65Dn mice. We speculate that this may be due to synergistic cellular and molecular effects between EE and EGCG since they share common functions such as neuroplasticity enhancement, antioxidant activity, anti-inflammatory function, neuroprotection, promotion of the non-amyloidogenic proteolytic pathway of APP, and Dyrk1A kinase activity inhibition. PCA highlighted the way in which variables contributed to the variance in our data sets. As discussed above, it identified a composite learning variable and demonstrated an increased variance along the learning process within all groups and identified some trisomic individuals as more prone to the effects of EE and the combined EE-EGCG treatment than others. Overall results suggest that the combination of EGCG and EE could be an efficient therapeutic strategy in older DS individuals although there may be a large heterogeneity in the clinical outcome (responders and non-responders).

## Funding

The laboratory of Mara Dierssen is supported by DIUE de la Generalitat de Catalunya (Grups consolidats SGR 2014/1125). This work was supported by Fondation Jérôme Lejeune (Paris, France), MINECO (SAF2013-49129-C2-1-R), CDTI (“Smartfoods”), and EU (Era Net Neuron PCIN-2013-060). The CRG is a Center of Excellence Severo Ochoa SEV-2012-0208. The CIBER of Rare Diseases is an initiative of the ISCIII. The laboratory of Cedric Notredame acknowledges the funding from the Spanish Ministry of Economy and Finance (MINECO), grant number BFU2011-28575. Silvina Catuara-Solarz received a FPI doctoral fellowship from Spanish Ministry of Economy and Finance (MINECO), SAF2010-16427; Jose Espinosa-Carrasco received the FI grant from Agència de Gestió d'Ajuts Universitaris i de Recerca (AGAUR) de la Generalitat de Catalunya.

### Conflict of interest statement

The authors declare that the research was conducted in the absence of any commercial or financial relationships that could be construed as a potential conflict of interest.
