# Supplementary material for: Principal Component Analysis of the Effects of Environmental Enrichment and (-)-epigallocatechin-3-gallate on Age-Associated Learning Deficits in a Mouse Model of Down Syndrome
Source: Front Behav Neurosci. 2015 Dec 11;9:330. doi: 10.3389/fnbeh.2015.00330 (PMC4675859; doi:10.3389/fnbeh.2015.00330)
Supplement: Supplementary file 1 [file DataSheet1.DOCX]

***Supplementary Material***

**Principal component analysis of the of environmental enrichment and (-)-epigallocatechin-3-gallate on age-associated learning deficits in a mouse model of Down syndrome**

**Silvina Catuara-Solarz^§1,3^, Jose Espinosa-Carrasco^§1,2,3^, Ionas Erb^§2,3^, Klaus Langohr^7,8^, Cedric Notredame^2,3^, Juan R González^5,6 *,^ Mara Dierssen^1,3,4*^**

^1^Cellular & Systems Neurobiology, Systems Biology Program, The Barcelona Institute of Science and Technology, Centre for Genomic Regulation (CRG), Dr. Aiguader 88, 08003 Barcelona, Spain

^2^Comparative Bioinformatics, Bioinformatics and Genomics Program, Barcelona Institute of Science and Technology, Centre for Genomic Regulation (CRG), Dr. Aiguader 88, 08003 Barcelona, Spain

^3^Universitat Pompeu Fabra (UPF), Barcelona, Spain

^4^Centro de Investigación Biomédica en Red de Enfermedades Raras (CIBERER), Spain

^5^Centre for Research in Environmental Epidemiology (CREAL)

^6^Centro de Investigación Biomédica en Red de Epidemiología y Salud Pública (CIBERESP), Spain

^7^Human Pharmacology and Clinical Neurosciences Research Group, Neurosciences Research Program, IMIM (Hospital del Mar Medical Research Institute), Barcelona, Spain

^8^Department of Statistics and Operations Research, Universitat Politècnica de Catalunya/BARCELONATECH, Barcelona, Spain

^§^These authors equally contributed

*** Correspondence:**

Mara Dierssen. Cellular & Systems Neurobiology, Systems Biology Program, Centre for Genomic Regulation (CRG), The Barcelona Institute of Science and Technology, Dr. Aiguader 88, 08003 Barcelona, Spain

[mara.dierssen@crg.eu](mailto:mara.dierssen@crg.eu)

Juan R. González. Centre for Research in Environmental Epidemiology (CREAL), Parc de Recerca Biomèdica de Barcelona (PRBB), Dr. Aiguader 88 - 08003 Barcelona, Spain

[jrgonzalez@creal.cat](mailto:jrgonzalez@creal.cat)

**Supplementary Figures and Tables**

**Supplementary Figure 1. Effects of EE, EGCG and EE-EGCG on thigmotactic behavior during the acquisition sessions in WT mice**. Fitted linear mixed model (represented as colored lines) and observations (dots) show mean ± SEM. ANOVA repeated measures with Tukey post-hoc comparisons corrected with BH; * p < 0.05, ** p < 0.01; WT non-treated group was considered as the reference in the comparisons. Both EE and combined treatment with EE-EGCG, but not EGCG promoted a significant reduction in the percentage of time in the periphery (thigmotatic behavior) across acquisition days in the WT mice.

**Supplementary Figure 2.** **Time spent in target quadrant among all the groups at the probe trial.** The figure shows panel boxplots of the distribution of the percentage of time in the target quadrant of all experimental groups in the removal session. In each boxplot, the horizontal line corresponds to group median, the box edges gives the 25^th^ and 75^th^ percentiles and the whiskers depict minimum and maximum values to a maximum of 1.5 times the interquartile distance from the box. More extreme values are individually plotted. Red dots indicate the values of each individual mouse. ANOVA shows no differences among the groups. This is probably due to the large within-group variance, depicted by the large distance between the box edges (25^th^ and 75^th^ percentiles).

**Supplementary Figure 3. Effects of EE, EGCG and EE-EGCG during the reversal sessions in WT and Ts65Dn mice.** Fitted linear mixed model is represented as colored lines and observations (dots) show mean ± SEM. **a. Latency to reach the platform** **in the reversal sessions.** Untreated Ts65Dn showed worse performance in finding the new target position of the platform when compared to WT, indicating poor cognitive flexibility. Even though there were no significant effects of any of the treatments on latency to reach the new platform positions neither in WT nor in Ts65Dn, both TS-EE (β = 11.78) and TS-EE-EGCG (β = 11.95) were qualitatively less different from untreated WT than untreated Ts65Dn mice (β = 18.46) taking into account the magnitude of the group differences by the model estimate (β). **b. Gallagher index during the reversal sessions.** Untreated Ts65Dn showed a trend towards an increased mean distance to the new platform position compared to WT, as shown by the Gallagher index. Neither of the treatments had effects on the Gallagher index on Ts65Dn or WT mice **c. Thigmotaxis**. Untreated Ts65Dn showed increased percentage of time spent in the periphery of the pool as compared to WT. Neither of the treatments had effects on the thigmotaxis on Ts65Dn or WT mice. ANOVA repeated measures with Tukey *post-hoc* comparisons corrected with BH; * *p* < 0.05, ** *p* < 0.01; WT and TS non-treated groups were considered as the references in the comparisons.

**Supplementary Figure 4: Projection of individual mice on the PCA unravels the phenotypic variability specific to learning.** The plot shows the 86 individuals projected on the space generated by the PCA of the experimental group medians. Colors depict experimental groups while numbers indicate the acquisition session of each animal. There is a large spread of the individuals within each of the experimental group and this variability increases along the acquisition sessions due to the effect of the different phenotypic capability of each animal to learn even when they belong to the same group.

**Supplementary Figure 5:** **WT mice groups manifested a homogenous learning behavior as reported by PC1. (A)** Density distribution of all WT groups (untreated and treated for the first and the second principal components of the PCA (PC1 and PC2). On the first acquisition session (left panel) all WT showed no significant difference between groups on this variable indicating a comparable basal performance of all animals. All WT mice reached high values of PC1 on the fifth acquisition session, at the end of the learning phase (right panel), denoting that they do not manifest any learning impairment. **(B)** Boxplots of the distribution of the first principal component for each WT group on the first and the fifth session of the acquisition phase. In each boxplot, the horizontal line corresponds to group median, the box edges gives the 25^th^ and 75^th^ percentiles and the whiskers depict minimum and maximum values to a maximum of 1.5 times the interquartile distance from the box. More extreme values are individually plotted.

**Supplementary Figure 6:** **Supervised PCA of the reversal sessions revealed the main direction of learning (cognitive flexibility) along the first principal component. (A)** Group performance (medians) in the new ordination space. Each trajectory represents an experimental group and connects the five reversal sessions labeled with its respective number. All group trajectories showed a progression towards positive values of the first principal component (PC1). In the case of the reversal, PC1 can be seen as a composite variable that recapitulates the cognitive flexibility of mice, i.e. the ability of mice to re-learn the position of the platform. PC2 show a more irregular distribution. **(B)** PCA of the variables, where arrows represent the direction of each variable in the PCA space. Arrows reaching the unit circle belong to variables that are well represented by the two principal components. In this case PC2 is not only contributed by speed but also by thigmotactic behavior. The direction that percentage in the periphery arrow point into is ordering the trajectories of mice. **(C)** Bar plots showing the percentage of explained variance for each principal component. Bars represent the contribution (%) of each variable to first and second principal components. The first principal component (left panel) can be interpreted as a composite (re-) learning variable. Learning-related variables had a similar contribution on PC1 ranging from 18% in the case of the distance to 17% in the case of the Whishaw index. Speed (right panel) and percentage in the periphery are the major contributors to PC2 with a contribution of 66% and 26% respectively.

**Supplementary Figure 7:** **The first principal component of the PCA (PC1) quantifies re-learning. (A)** Density distribution of all Ts65Dn groups for the first and the second principal components of the PCA (PC1 and PC2). PC1 quantifies mice cognitive flexibility, i.e. the ability of mice to re-learn the new location of the platform. Ts65Dn mice showed lower PC1 values during the first reversal session (left panel) than during the third reversal session, showing an increased performance across sessions. **(B)** Boxplots of the distribution of the first principal component for each Ts65Dn group on the first and the third session of the reversal phase. In each boxplot, the horizontal line corresponds to group median, the box edges gives the 25th and 75th percentiles and the whiskers depict minimum and maximum values to a maximum of 1.5 times the interquartile distance from the box. More extreme values are individually plotted. TS-EE-EGCG reached higher values on the composite learning variable than the other Ts65Dn mice on the third reversal session, although without reaching statistical significance (*p*-value = 0.08 for comparison with the untreated group).

**Supplementary Table 1. Permutation test results of significant pairwise comparisons for the PC1, on the first acquisition session, sorted in ascending p-value order.**

| **Comparison** | **P (10000 permutations)** |
| --- | --- |
| TS-EGCG vs. WT-EEEGCG | <0,001 |
| TS-EGCG vs. WT-EE | 0,003 |
| TS-EGCG vs. WT | 0,003 |
| TS-EE vs. WT-EEEGCG | 0,004 |
| TS vs. WT-EEEGCG | 0,012 |
| TS-EEEGCG vs. WT-EEEGCG | 0,012 |
| TS-EGCG vs. WT-EGCG | 0,012 |
| TS-EE vs. WT-EE | 0,020 |
| TS-EE vs. WT | 0,020 |
| TS-EEEGCG vs. TS-EGCG | 0,030 |
| TS vs. WT-EE | 0,045 |
| TS vs. WT | 0,047 |

**Supplementary Table 2. Permutation test results of significant pairwise comparisons for the PC1, on the fifth acquisition session, sorted in ascending p-value order.**

| **Comparison** | **P (10000 permutations)** |
| --- | --- |
| TS vs. WT-EE | <0.001 |
| TS vs. WT-EEEGCG | <0.001 |
| TS vs. WT-EGCG | <0.001 |
| TS-EGCG vs. WT-EE | <0.001 |
| TS vs. WT | 0,001 |
| TS-EE vs. WT-EE | 0,002 |
| TS-EGCG vs. WT-EEEGCG | 0,002 |
| TS-EGCG vs. WT-EGCG | 0,002 |
| TS-EE vs. WT-EGCG | 0,007 |
| TS-EGCG vs. WT | 0,008 |
| TS vs. TS-EEEGCG | 0,008 |
| TS-EE vs. WT-EEEGCG | 0,009 |
| TS-EE vs. WT | 0,025 |
| TS-EEEGCG vs. TS-EGCG | 0,036 |

**Supplementary Table 3. Permutation test results of significant pairwise comparisons for the PC1, on the first reversal session, sorted in ascending p-value order.**

| **Comparison** | **P (10000 permutations)** |
| --- | --- |
| WT vs. TS-EGCG | <0.001 |
| WT-EE vs. TS-EGCG | <0.001 |
| TS-EGCG vs. WT-EEEGCG | 0,002 |
| WT vs. TS-EE | 0,002 |
| WT-EE vs. TS-EE | 0,004 |
| WT-EGCG vs. TS-EGCG | 0,006 |
| WT vs. TS | 0,007 |
| TS-EE vs. WT-EEEGCG | 0,019 |
| TS vs. WT-EE | 0,022 |
| TS-EE vs. WT-EGCG | 0,037 |

**Supplementary Table 4. Permutation test results of significant pairwise comparisons for the PC1, on the third reversal session, sorted in ascending p-value order.**

| **Comparison** | **P (10000 permutations)** |
| --- | --- |
| WT-EE vs. TS-EE | 0,000 |
| TS vs. WT-EE | 0,000 |
| TS-EE vs. WT-EEEGCG | 0,000 |
| TS vs. WT-EEEGCG | 0,000 |
| WT vs. TS | 0,001 |
| WT vs. TS-EE | 0,001 |
| TS-EGCG vs. WT-EEEGCG | 0,001 |
| WT-EE vs. TS-EGCG | 0,003 |
| TS vs. WT-EGCG | 0,007 |
| TS-EE vs. WT-EGCG | 0,007 |
| WT vs. TS-EGCG | 0,009 |
| WT-EGCG vs. TS-EGCG | 0,031 |
| WT-EGCG vs. WT-EEEGCG | 0,031 |
| WT-EEEGCG vs. TS-EEEGCG | 0,039 |
