# Supplementary figures and images for: Principal Component Analysis of the Effects of Environmental Enrichment and (-)-epigallocatechin-3-gallate on Age-Associated Learning Deficits in a Mouse Model of Down Syndrome
Source: Front Behav Neurosci. 2015 Dec 11;9:330. doi: 10.3389/fnbeh.2015.00330 (PMC4675859; doi:10.3389/fnbeh.2015.00330)

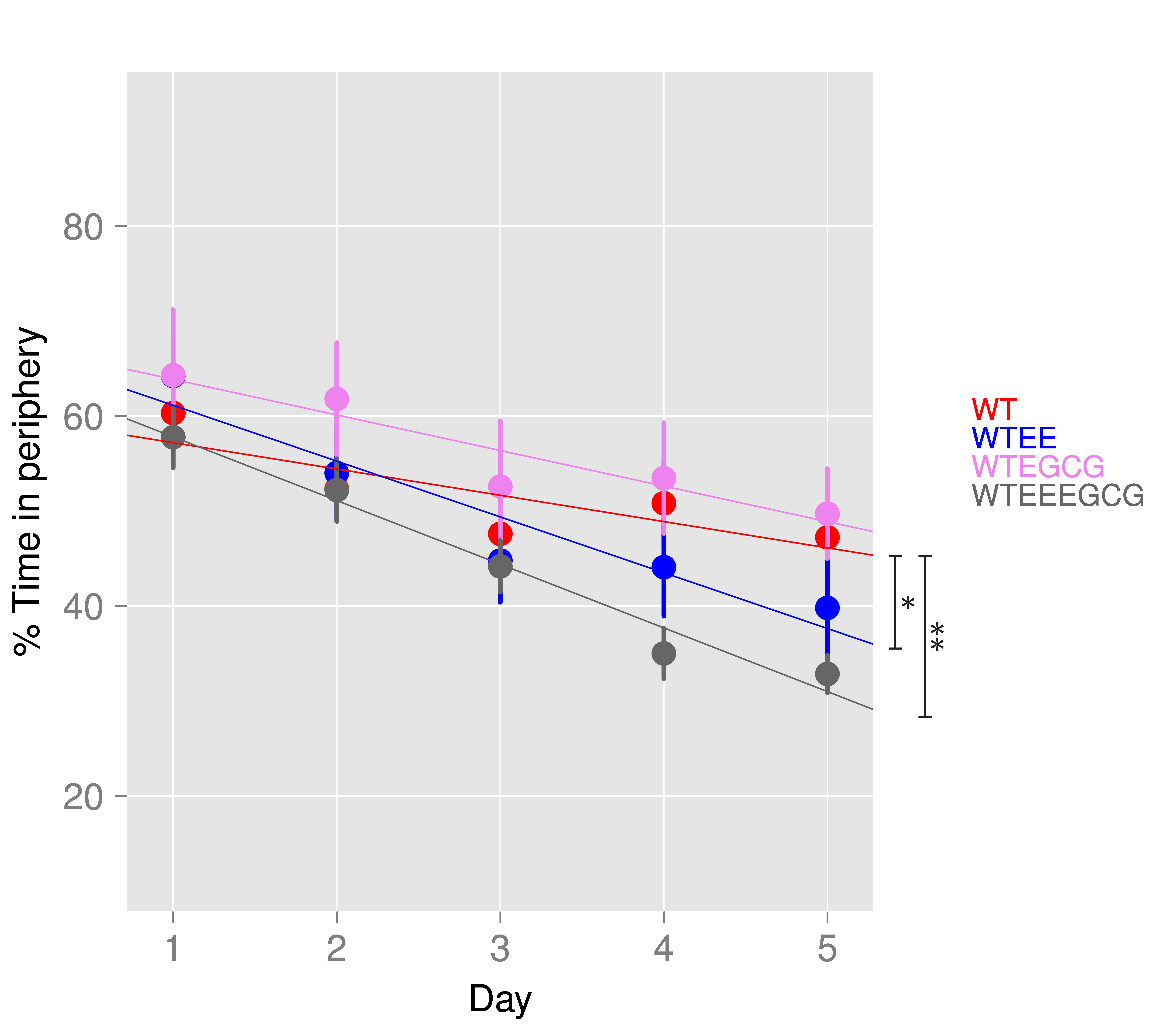

Supplement: Supplementary file 2 [file Image1.JPEG]

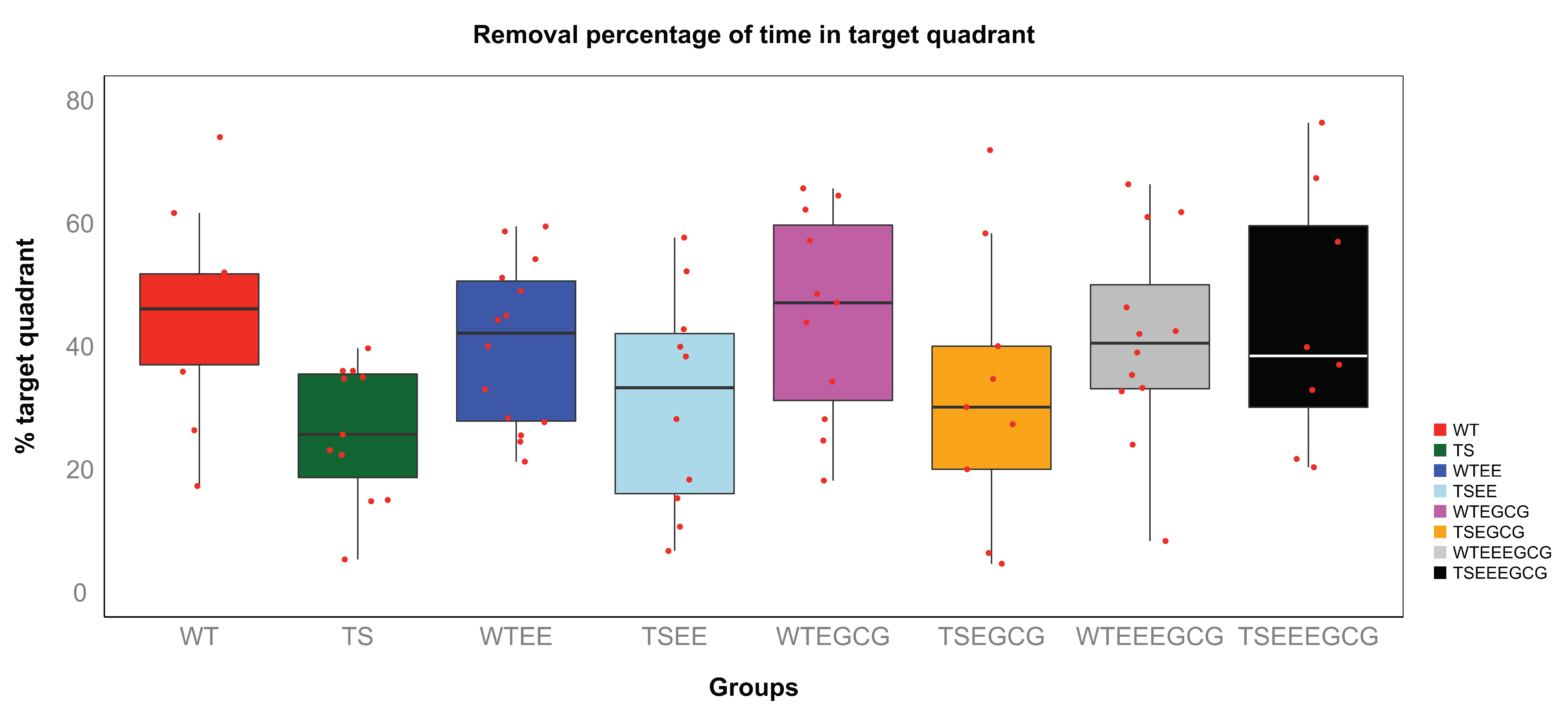

Supplement: Supplementary file 3 [file Image2.JPEG]

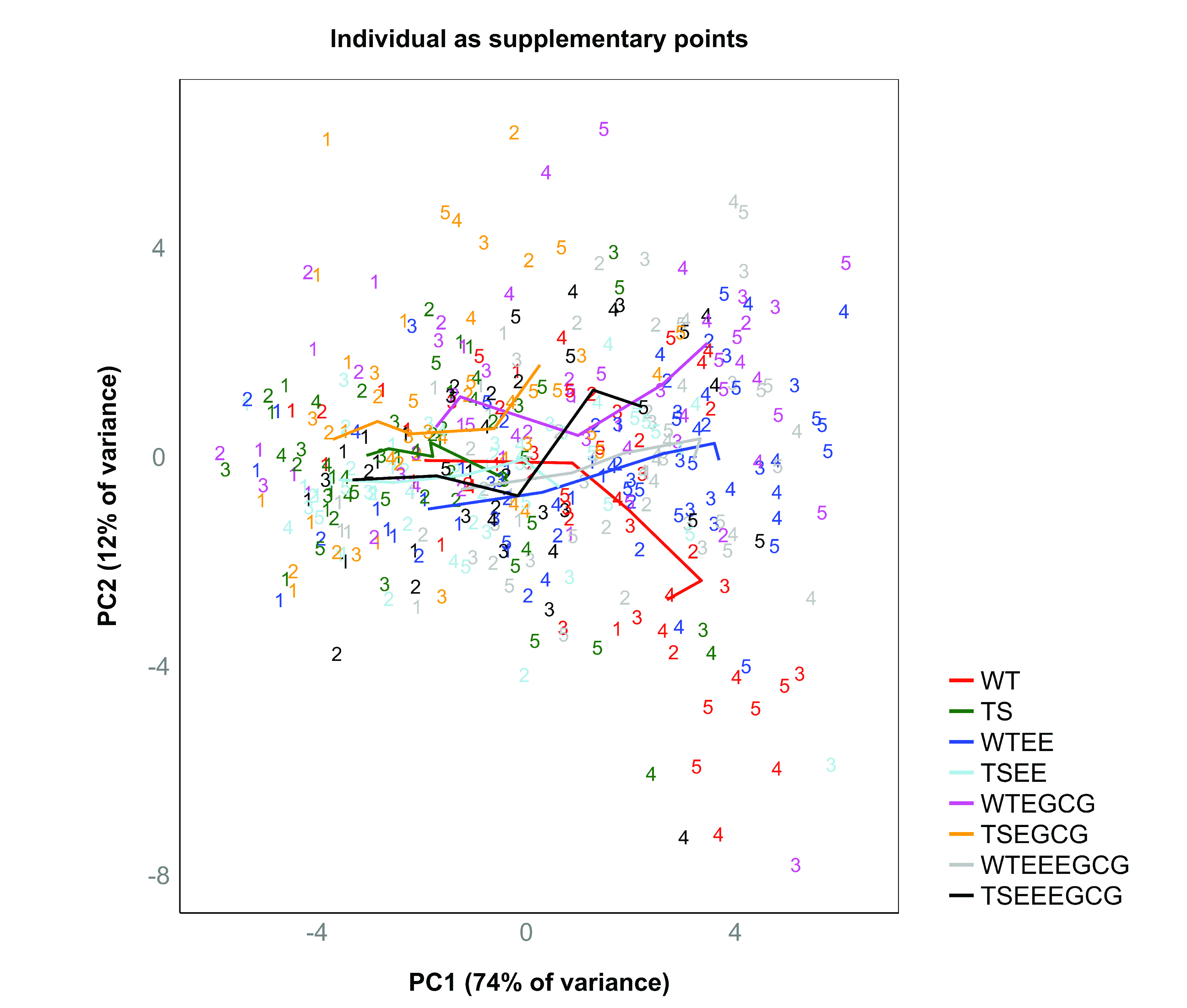

Supplement: Supplementary file 5 [file Image4.JPEG]

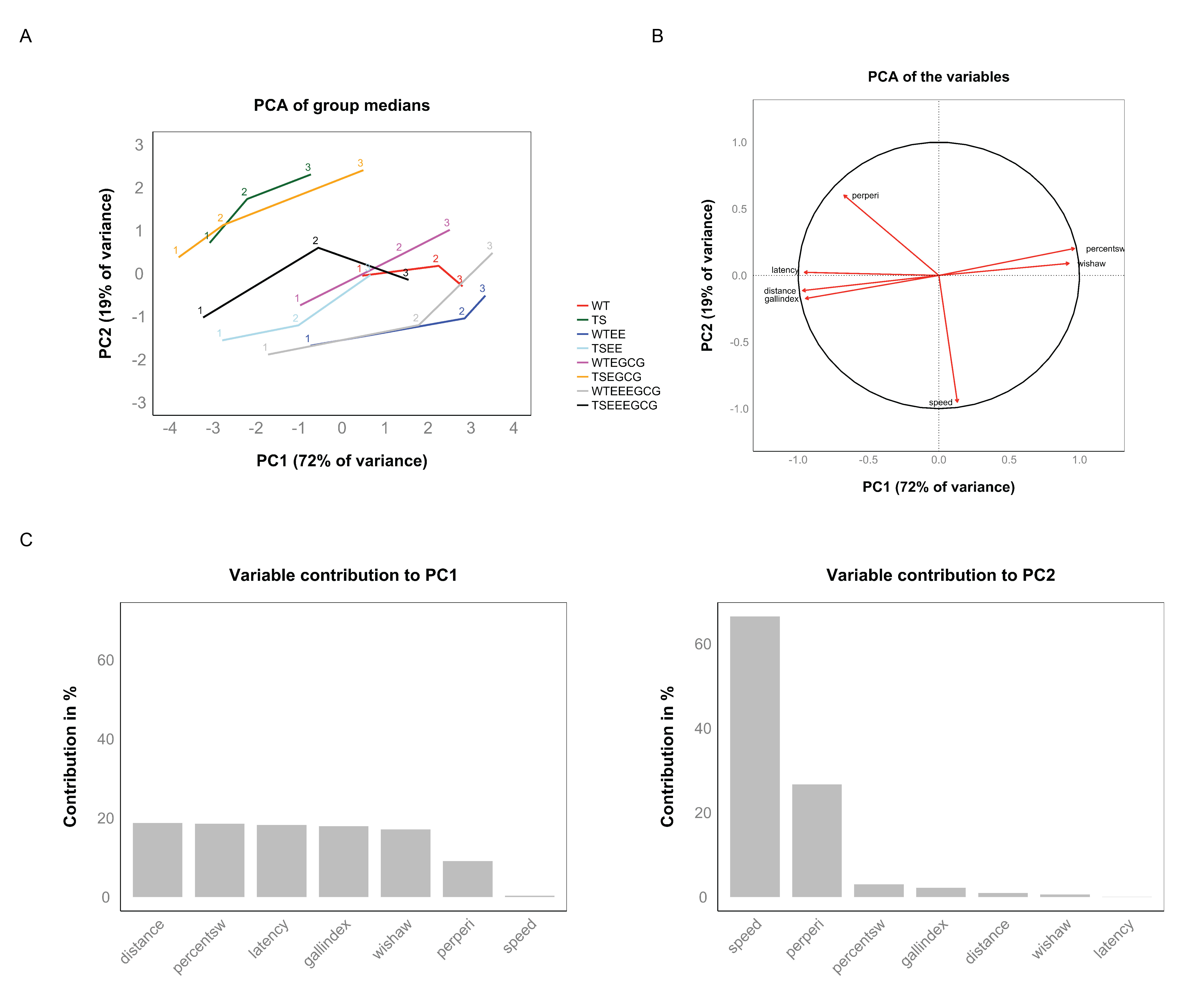

Supplement: Supplementary file 7 [file Image6.JPEG]
